# Specific Inhibition of Soluble γc Receptor Attenuates Collagen-Induced Arthritis by Modulating the Inflammatory T Cell Responses

**DOI:** 10.3389/fimmu.2019.00209

**Published:** 2019-02-08

**Authors:** Byunghyuk Lee, Yuna Jo, Geona Kim, Laraib Amir Ali, Dong Hyun Sohn, Seung-Geun Lee, Kiseok Kim, Euisu Shin, Sung Ho Ryu, Changwan Hong

**Affiliations:** ^1^Department of Anatomy, Pusan National University School of Medicine, Yangsan, South Korea; ^2^Department of Microbiology and Immunology, Pusan National University School of Medicine, Yangsan, South Korea; ^3^Division of Rheumatology, Department of Internal Medicine, Pusan National University School of Medicine, Pusan National University Hospital, Busan, South Korea; ^4^Aptamer Sciences Inc., POSTECH Biotech Center, Pohang, South Korea; ^5^Department of Life Sciences, Pohang University of Science and Technology, Pohang, South Korea

**Keywords:** soluble common gamma chain, aptamer, collagen-induced arthritis, Th17, IL-2

## Abstract

IL-17 produced by Th17 cells has been implicated in the pathogenesis of rheumatoid arthritis (RA). It is important to prevent the differentiation of Th17 cells in RA. Homodimeric soluble γc (sγc) impairs IL-2 signaling and enhances Th17 differentiation. Thus, we aimed to block the functions of sγc by inhibiting the formation of homodimeric sγc. The homodimeric form of sγc was strikingly disturbed by sγc-binding DNA aptamer. Moreover, the aptamer effectively inhibited Th17 cell differentiation and restored IL-2 and IL-15 signaling impaired by sγc with evidences of increased survival of T cells. sγc was highly expressed in SF of RA patients and increased in established CIA mice. The therapeutic effect of PEG-aptamer was tested in CIA model and its treatment alleviated arthritis pathogenesis with impaired differentiation of pathogenic Th17, NKT1, and NKT17 cells in inflamed joint. Homodimeric sγc has pathogenic roles to exacerbate RA progression with differentiation of local Th17, NKT1, and NKT17 cells. Therefore, sγc is suggested as target of a therapeutic strategy for RA.

## Introduction

The common gamma chain (γc) is a cytokine receptor subunit that is shared by the γc family cytokines and is composed of IL-2,−4,−7,−9,−15, and−21 (1). It has been described that the signals of γc cytokines are essential to the proliferation, differentiation, homeostasis, and activities of cells in the innate and adaptive immune systems (2, 3), showing that the genetic mutations or deficiencies of γc result in fetal immunodeficiency disorder in human and mice (4, 5). A soluble form of γc (sγc) has recently been reported, showing that sγc is generated by alternative splicing and plays an antagonistic role in γc cytokine signaling (6). The secreted sγc forms a homodimeric structure that binds to IL-2Rβ with a higher affinity than the monomer sγc (6). However, the dimerization mechanisms of sγc have not been fully elucidated. IL-2 has important roles in controlling the balance between helper T 17 (Th17) cells and regulatory T (Treg) cells. Although IL-2 is essential for the differentiation and functions of Treg cells (7, 8), the Th17 differentiation is negatively regulated by IL-2 (9, 10). IL-2 deficiency leads to increased pro-inflammatory Th17 cell populations (9), playing pivotal roles in the pathogenesis of several autoimmune diseases, including RA, which is characterized by chronic inflammation of the joints and irreversible joint damage. The detection of Th17 cell population in peripheral blood and SF of RA patients can be an indicator of inflammatory activity (11–13). Thus, the regulation of IL-2 signaling will provide an important clue for the management of RA pathogenesis.

IL-17 has been demonstrated as a key factor in collagen-induced arthritis (CIA) (14). IL-17 leads to the upregulation of receptor activator of nuclear factor kappa-B ligand (RANKL), activating osteoclast, and resulting in bone resorption (15). Moreover, it also induces synovial macrophages to produce TNF-α and IL-1 involved in the formation of osteoclasts. IL-17 is mainly produced by Th17 cells, but is also produced by other types of immune cells, including CD8^+^ T cells (16), innate lymphoid cells (ILCs) (17), and natural killer T cells (NKT) (18). Several studies have reported that NKT cells had a pathogenic role in CIA mouse models with an increased number of IL-17 producing NKT cells (19–21).

Since the sγc promotes the differentiation of Th17 cells by inhibiting IL-2 signaling, it can also exacerbate the severity of Th17 cell-mediated inflammation, as demonstrated in experimental autoimmune encephalomyelitis (EAE) model (6). Indeed, sγc overexpressing and sγc-deficient mice showed a conflicting prognosis of EAE disease (6). Importantly, it has been previously reported that the level of sγc expression is highly detected in autoimmune diseases, including inflammatory bowel disease (IBD) (22) and RA (23, 24). Thus, we thought that the high level of sγc may contribute to the aggravation of inflammatory autoimmune diseases and wanted to target the dimerization of sγc to control its function by introducing an aptamer.

Aptamer is a small, single-stranded (ss) DNA or RNA oligonucleotide and an alternative strategy for binding to specific target molecules with high affinity and specificity (25). Aptamers have striking advantages over monoclonal antibodies, in that they are stable, chemically synthesized at a low cost, and have no evidence of significant immunogenicity, with a high binding affinity (25, 26). Given its wide array of advantages, it is considered a prospective candidate for a novel diagnostic or therapeutic approach to cancer, viral infection, and inflammatory diseases (27). Aptamers are generally obtained by a standard procedure; the systematic evolution of ligands by exponential enrichment (SELEX) to select high affinity aptamers that are specific for target molecules or cells (25, 28).

Here, we showed that the generation of sγc homodimer is elicited by a disulfide bond and developed specific aptamers that inhibit the dimerization of sγc. Moreover, we found that the enhanced level of sγc in CIA mice promoted the differentiation of Th17, NKT1, and NKT17 cells; however, these pathogenic roles of sγc were suppressed by sγc-aptamer. Taken together with the elevated level of sγc in human RA patients, these studies suggested that the homodimer form of sγc may contribute to the exacerbation of the pathogenesis of CIA and blockade of the function of sγc conceivably improves RA.

## Materials and Methods

### Patients

Consecutive patients clinically diagnosed with rheumatoid arthritis (RA) or osteoarthritis (OA) were included. All patients with RA (*n* = 10) met the 2010 American College of Rheumatology/European League Against Rheumatism classification criteria for rheumatoid arthritis (29) and all patients with OA (*n* = 9) fulfilled the clinical criteria of the American College of Rheumatology for knee OA (30). For RA patients, the mean (±SD) age was 58.6 (±11.1) years and all were female. All patients with RA were treated with at least one disease modifying anti-rheumatic drugs. The study was approved by the Research and Ethical Review Board of the Pusan National University (PNU) Hospital (IRB 1608-015-044). All study subjects provided written informed consent in accordance with the principles of the Declaration of Helsinki.

### Animals

DBA/1 mice were obtained from Orient Bio, South Korea. All animal experiments and protocols were approved by the PNU Institutional Animal Care and Use Committee (PNU-2017-1605) and were housed in a specific pathogen-free animal facility at PNU School of Medicine.

### Modified Systematic Evolution of Ligands by Exponential Enrichment (SELEX)

The advanced SELEX technology was used as previously described (31). In brief, aptamers were selected from a ssDNA library containing a 40-nucleotide randomized region, in which 5-(N-benzylcarboxyamide)-20-deoxyuridine (Bz-dU) or 5-(N-naphthylcarboxyamide)-20-deoxyuridine (Nap-dU) was substituted for dT. The oligonucleotides contained a central randomized region of 40 nucleotides, which were flanked by two conserved flanking regions with 17 nucleotides (5′-CGAGCGTCCTGCCTTTG-40N-CACCGACAGCCACCCAG-3′). The SELEX process was performed at 37°C. A mixture of aptamer library dissolved in a buffer solution was heated at 95°C for about 5 min and then was slowly cooled to 37°C for re-folding. The aptamer library was pre-incubated with Hexa-his tag magnetic bead (Invitrogen) to eliminate non-specific binder. In addition, the aptamer library binding control γc-extracellular domain (ED) was also removed from each pool by negative selection. The aptamer library in supernatant was incubated with purified sγc (including the C-terminal CLQFPPSRI), and then the target protein was isolated by Dynabeads (ThermoFisher). Aptamers bound to the target protein were eluted and amplified via PCR reaction. The resulting aptamers were used in the next SELEX round. Truncated or modified aptamers with 5′-PEG and 3′-inverted dT were obtained from Aptamer Science Inc.

### Cloning and Sequencing of Selected Aptamers

After 8 rounds of SELEX, the eluted aptamers were amplified by QPCR using primers, and then cloned into TA cloning Kit and the cloned parts were sequenced (Solgent). Sequences of the selected aptamers were aligned using the “aptamer motif searcher,” an in-house program of POSTECH Aptamer Initiative, and a pattern analysis was performed. The secondary structures of aptamers were predicted by the mfold Web Server (http://unafold.rna.albany.edu).

### Binding Affinity Assays

The aptamer–protein equilibrium dissociation constants (Kd) were determined via the nitrocellulose-filter binding method (32). For all binding assays, aptamers were dephosphorylated using alkaline phosphatase, 5-end labeled using T4 polynucleotide kinase (New England Biolab) and [^32^P]-ATP (Amersham Pharmacia Biotech). Direct binding assays were carried out by incubating a ^32^P-labeled aptamer at a concentration of <10 pM and protein at concentrations ranging from 10 pM to 100 nM in a selection buffer. The fraction of bound aptamer was quantified with a PhosphorImager (Fuji FLA-5,100 Image Analyzer). Raw binding data were corrected for non-specific background binding of radiolabeled aptamer to the nitrocellulose filter.

### Immunoprecipitation and Western Blot

The sγc in supernatants of cultured cells were immunoprecipitated with α-mouse IL-2Rγ antibody (R&D systems) and protein A/G agarose beads (Santa Cruz Biotechnology). Immunoprecipitates were resolved by SDS-PAGE (Novex) under reducing by dithiothreitol (DTT) or non-reducing conditions and transferred to a polyvinylidene difluoride (PVDF) membrane (Amersham Biosciences). Blots were incubated with biotinylated α-mouse IL-2Rγ antibody (R&D systems), followed by HRP-conjugated streptavidin (BioLegend). The membranes were developed by enhanced chemiluminecence (ECL) reagents (GE Healthcare). The bands were detected using LAS-3000 Imaging system (Fujifilm).

### Plasmid Construction and Mutagenesis

The modification of sγc gene was performed by designing the reverse primers in two ways. One was to alter the amino acid (a.a.) sequence of cysteine (Cys) to alanine (Ala) at C-terminal residues (sγcC255A); the other was to delete the a.a. sequences of all 9 proteins at C-terminal residues (sγcΔ255-263). Primer sequences are as follows; 5′-ATG TTG AAA CTA TTA TTG TCA-3′ for sγc forward, 5′-GAT TCT TGA TGG GGG GAA-3′ for sγc reverse, 5′-GAT TCT TGA TGG GGG GAA TTG GAG GGC TTC-3′ for sγcC255A reverse, 5′-TTC CTC TAC AGT ATG ACT-3′ for sγcΔ255-263 reverse.

### Transient Transfection

Human embryonic kidney (HEK) 293T cells were used for transient transfection. The respective DNA constructs were mixed gently with Lipofectamine™ 2000 (Invitrogen) and incubated at room temperature (RT) for 15 min. The mixture was incubated on HEK 293T cells for 6 h. In order to examine an effect of aptamers, full length, truncated, or scramble aptamers were treated 6 h after transfection.

### *In vitro* Stimulation With IL-2 or IL-15

LN T cells were incubated *in vitro* with 10 ng/ml recombinant IL-2 (Peprotech) or 20 ng/ml recombinant IL-15 (Peptrotech) in the presence of sγc, with or without sγc-aptamer. LN T cells were stained for intracellular Bcl-2 expression, using the Foxp3 staining kit in accordance with the manufacturer's instructions (eBioscience). Active-caspase3 induction was determined using CaspGLOW™ fluorescein active-caspase3 staining kit (eBioscience).

### *In vitro* T Cell Differentiation

For *in vitro* differentiation, the condition media were generated in the following manner: IL-2 (20 ng/ml; Peprotech), IL-12 (100 ng/ml; eBioscience), and α-IL-4 (10 ug/ml; BioLegend) for Th1. IL-2 (20 ng/ml; Peprotech), IL-4 (100 ng/ml; Peprotech) and α-IFNγ (10 ug/ml; BioLegend) for Th2. IL-6 (30 ng/ml; BD), TGF-β1 (50 ng/ml; Peprotech), α-IFNγ (10 ug/ml; BioLegend) and α-IL-4 (10 ug/ml; BioLegend) for Th17. 100 nM of respective aptamers were treated in differentiation of CD4^+^ T cells.

### Flow Cytometry

Antibodies with the following specificities were used for staining: CD4 (GK1.5) from eBioscience; TCRβ (H57-597), CD8 (53-6.7), IFNγ (XMG1.2), IL-4 (11B11), Bcl-2 (BCL/10C4), T-bet (4B10), PLZF (9E12) from BioLegend; IL-17 (TC11-18H10) from BD Bioscience. Fluorochrome-conjugated CD1d tetramers loaded with PBS-567 were obtained from NIH tetramer facility (Emory University, Atlanta, GA). Intranuclear PLZF and T-bet were detected using the Foxp3 staining kit as the manufacturer's instructions (eBioscience).

### ELISA

The sγc level in serum of mice were detected by a sandwich ELISA with γc-specific polyclonal antibody (R&D system) as capture antibody and biotin-conjugated γc-specific monoclonal antibody (R&D system) as detection antibody. The human sγc level from SF of RA or OA patients were measured with human γc-specific monoclonal antibody (R&D Systems) as capture antibody and biotin-conjugated human γc-specific polyclonal antibody (R&D Systems) as detection antibody. Concentration of sγc was calculated using the standard curve of recombinant sγc protein.

### Induction of CIA and Aptamer Treatment *in vivo*

Mice were injected intradermally (i.d.) at the base of the tail with chicken type II collagen (CII, Chondrex) emulsified in Freund's complete adjuvant (CFA). The immunized mice were boosted at 2 weeks after the initial immunization. Arthritis severity was scored from 0 to 4, with 0 indicating no evidence of erythema and swelling; 1 indicating mild erythema and swelling confined to the tarsals or ankle joint; 2 indicating mild erythema and swelling from the ankle to the tarsals; 3 indicating moderate erythema and swelling from ankle to the tarsals; and 4 indicating severe erythema and swelling encompassing the ankle, foot, and digits. The scores of all four paws were summed to obtain the arthritis score. For the treatment of aptamer, mice were injected intraperitoneally (i.p.) with PEG and idT-conjugated aptamer (PEG-aptamer) 6 times at boosting point (BP) or after onset of disease (AO).

### Histological Analysis

Mouse limbs were removed, fixed in 10% neutral buffered formalin, and decalcified. The decalcified limbs were embedded in paraffin and sections were prepared. The sections were stained with H&E and Safranin O to measure the inflammation and the loss of proteoglycans using standard procedures, respectively. Image analysis was performed by the Axio Scan Z1 (Carl Zeiss MicroImaging, Germany). H&E and safranin O staining was scored with a semiquantitative scoring system from 0 to 3, where 0 means no inflammation and 3 indicates severe inflammation for H&E and 0 indicates no proteoglycan loss and 3 represents complete loss of proteoglycan for safranin O.

### Micro-CT

Three-dimensional reconstruction images of pre-CIA and CIA-established mice were obtained by microfocal computed tomography (micro-CT, NFR Polaris-G90) at the day 33 of disease, according to the manufacturer's instructions.

### Statistical Analysis

Differences between the groups were examined with Student's two-tailed *t*-test or one-way ANOVA using Prism software (GraphPad). *P*-values of <0.05 were considered statistically significant. ^*^p < 0.05, ^**^p < 0.01, ^***^p < 0.001, and ns (not significant).

## Results

### sγc Specific Binding Aptamers Are Selected by Modified SELEX

**Figure 1:**
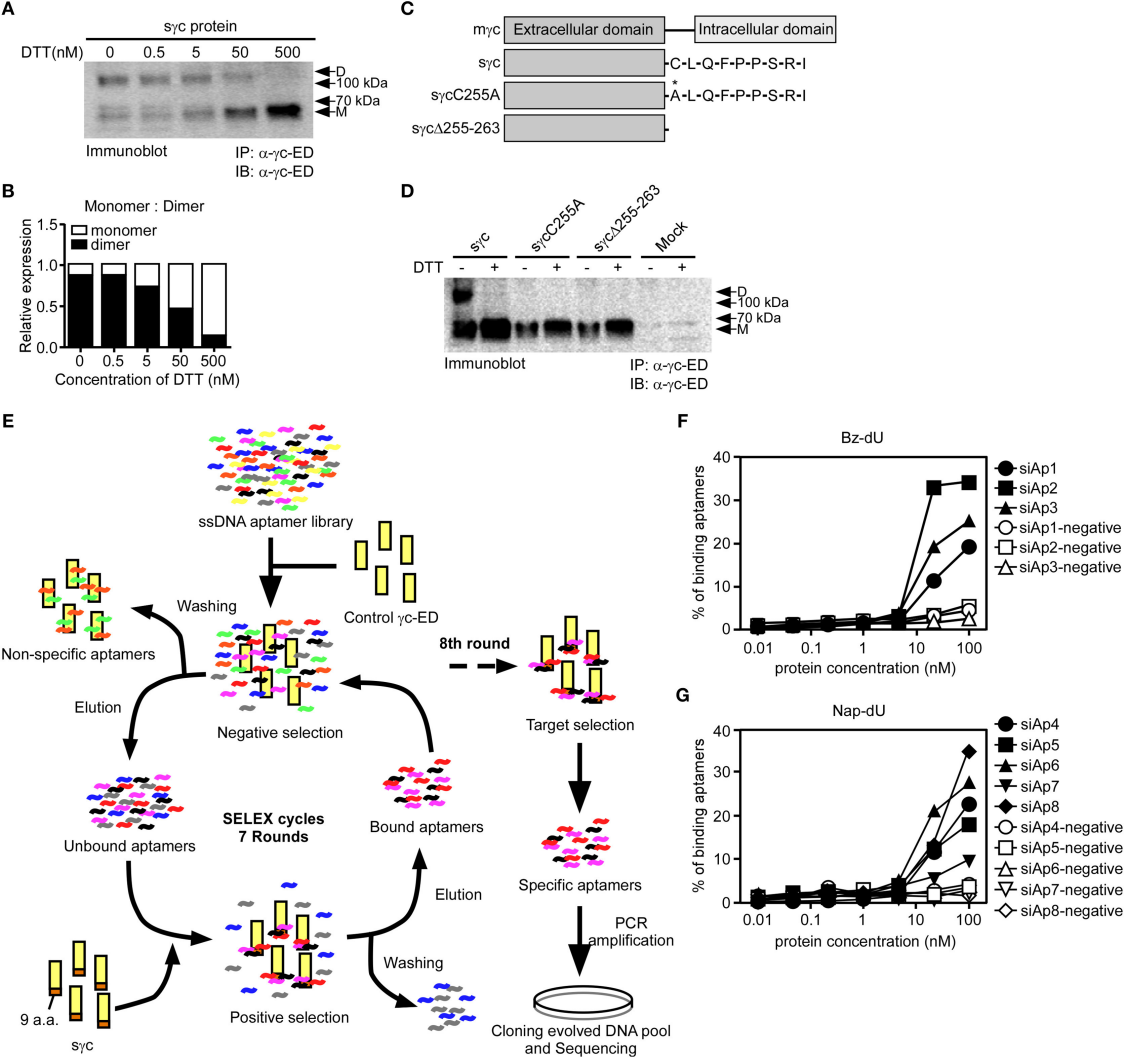
Cys residues in dimerization of sγc and selection of aptamers by SELEX. **(A)** Culture supernatant of sγc expressing HEK293 T cells were immunoprecipitated and immunoblotted for sγc proteins using α-γc-ED. Immunoprecipitates were reduced by DTT in a dose-dependent manner. Relative expression of monomer and dimer sγc of **(A)** was demonstrated in **(B)**. **(C)** Constructs of sγc, sγcC255A and sγcΔ255-263 were generated as depicted. **(D)** Culture supernatant of sγc expressing HEK293 T cells were immunoprecipitated and immunoblotted for sγc proteins using α-γc-ED. Immunoprecipitates were resolved by SDS-PAGE under reducing (DTT+) or non-reducing conditions (DTT-). The supernatant of HEK293 T cells (Mock) was used as negative control. **(E)** Scheme of selection of 9 a.a.-specific aptamers by SELEX. **(F,G)** Binding affinity assays were performed with Bz-dU **(F)** or Nap-dU **(G)** aptamers and the indicated concentration of sγc protein. D, dimer. M, monomer.

According to a previous study (6), sγc is present in the disulfide-linked homodimeric form, which is more functional than the monomeric form. Since sγc is generated by alternative splicing, it has a new C-terminal epitope, composed of 9 a.a., including Cys residue (6). It has been known that Cys residues are required to form disulfide bonds (33). To investigate whether Cys residues are essential in formation of homodimeric sγc, DTT was serially treated. The formation of dimeric sγc was dose-dependently inhibited, and the monomeric forms were increased by DTT (Figures 1A,B). To further confirm, we generated two mutant constructs: the Cys is substituted with Ala (sγcC255A); and 9 a.a. are totally deleted (sγcΔ255-263), as shown in Figure 1C. We found that sγcC255A and sγcΔ255-263 were not expressed as dimer forms, whereas the WT sγc was expressed as dimer forms and monomerized by DTT (Figure 1D). These data indicate that the dimerization of sγc requires Cys residues. To modulate the functional activity of sγc, we targeted 9 a.a. of sγc with specific aptamers to disrupt the formation of homodimeric sγc. Specific aptamers of 9 a.a. was screened by the SELEX, as described in Figure 1E. 3 ssDNA Bz-aptamers (Figure 1F) and 5 ssDNA Nap-aptamers (Figure 1G) were selected with low K_d_ and high B_max_ value, suggesting maximum binding to the target proteins (Table S1).

### sγc Inhibiting Aptamer (siAp) Blocks Dimerization of sγc and Impairs Th17 Differentiation

**Figure 2:**
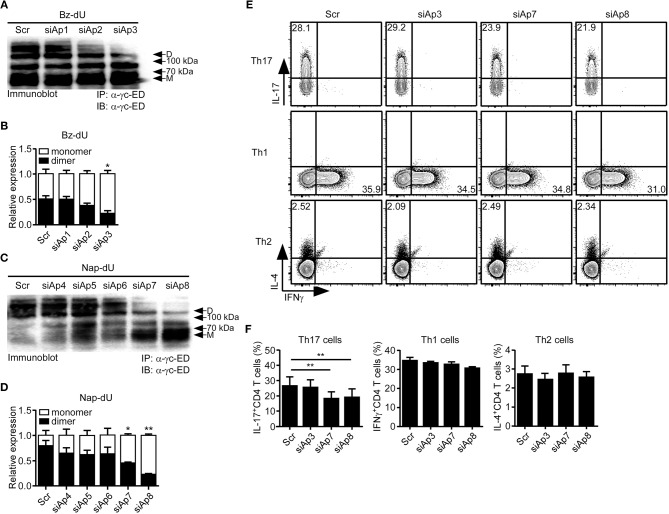
Effect of siAps in the dimerization of sγc and Th17 cell differentiation. **(A–D)** Culture supernatant of sγc expressing HEK293 T cells that was treated with aptamers were immunoprecipitated and immunoblotted for sγc proteins using α-γc-ED. Representative data of three independent experiments are shown for Bz-dU **(A)** and for Nap-dU **(C)**. Bar graphs show a summary of three independent experiments of the relative expression of monomeric and dimeric sγc for Bz-dU (**B**, means and SEM) and for Nap-dU (**D**, means and SEM). Scramble aptamer was used as the negative control. **(E,F)** Naïve CD4^+^ T cells were cultured with siAps under Th differentiation conditions. IFNγ, IL-4, and IL-17 expressions were assessed by intracellular staining. **(E)** Contour plots are representative of three independent experiments. **(F)** Bar graphs show a summary of three independent experiments (means and SEM). ^*^P <0.05; ^**^P <0.01. Scr, scramble. D, dimer. M, monomer.

First, to test which aptamers effectively inhibit the dimerization of sγc, we treated the aptamer candidates to sγc-producing HEK293T cells. We found that siAp3 (Figures 2A,B), siAp7 and siAp8 (Figures 2C,D) efficiently disturbed the formation of dimeric sγc. Since sγc enhanced Th17 cell differentiation of CD4^+^ T cells (6), the functional activity of siAps was tested in Th17 differentiation. siAp3 did not affect Th17 differentiation. On the other hand, siAp7 and siAp8 significantly inhibited Th17 cell differentiation, whereas there were no changes in Th1 and Th2 differentiations (Figures 2E,F). These results indicate that siAp7 and siAp8 are effective aptamers for the suppression of sγc dimerization and Th17 differentiation.

### Core Sequences of siAp Are Sufficient to Interrupt Dimeric Formation of sγc

**Figure 3:**
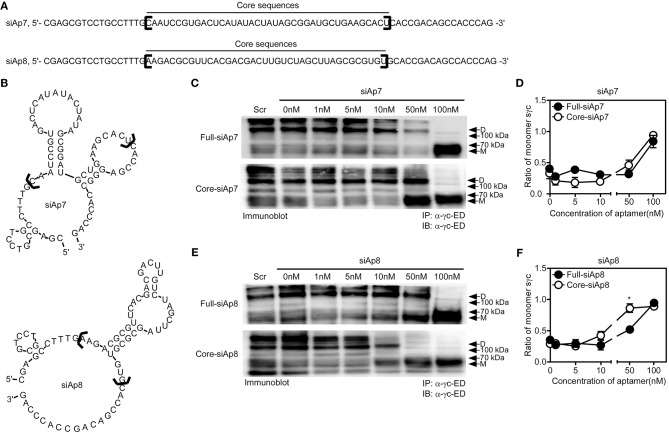
Sequence analysis of siAps. **(A,B)** The secondary stem-loop motifs were predicted with the full sequences of siAp7 and siAp8. Core sequences were marked with black brackets in **(A,B)**. **(C–F)** HEK293 T cells were transfected with sγc construct. After 6 h of transfection, the media was changed with full-siAp or core-siAp containing media. Relative expression of monomeric and dimeric sγc was detected by immunoprecipitation and immunoblot assay. Representative data of three independent experiments are shown for siAp7 **(C)** and for siAp8 **(E)**. **(D,F)** Line graph shows a summary of three independent experiments of the ratio of monomeric sγc against dimeric sγc (mean and SEM). Scramble aptamer was used as the negative control. ^*^P < 0.05. D, dimer. M, monomer.

Aptamer has target-non-specific arms and target-specific core sequences (25). With the sequences of siAp7 and siAp8 (Figure 3A), we predicted the secondary structures (Figure 3B). Since the stem-loop structures organized by core sequences are generally functional (34), we examined the functional capacity of core sequences in dimeric formation of sγc. We generated the truncated forms of siAp (core-siAp) with core sequences and tested the antagonistic capability of core-siAp compared with full-siAp. Full-siAp7 only induced the monomerization of sγc at a concentration of 100 nM (Figures 3C top**,D**). Although the formation of homodimeric sγc was partially impeded at 50 nM core-siAp7 (Figure 3C bottom), it was not significant (Figures 3C,D). Core-siAp8 was more functional than full-siAp8 with the fact that homodimeric sγc was completely monomerized in 50 nM core-siAp8 compared with full-siAp8 (Figures 3E,F). In comparison of siAp7 and siAp8, siAp8 was more functional than siAp7, as shown that siAp8 more effectively induced monomerization of sγc even in lower dose than siAp7 (Figures 3D,F). Collectively, we found that the core-siAp is sufficiently efficient in suppressing the formation of homodimeric sγc, and siAp8 is more effective than siAp7.

### Impaired IL-2 and IL-15 Signaling by sγc Is Restored by siAp8

**Figure 4:**
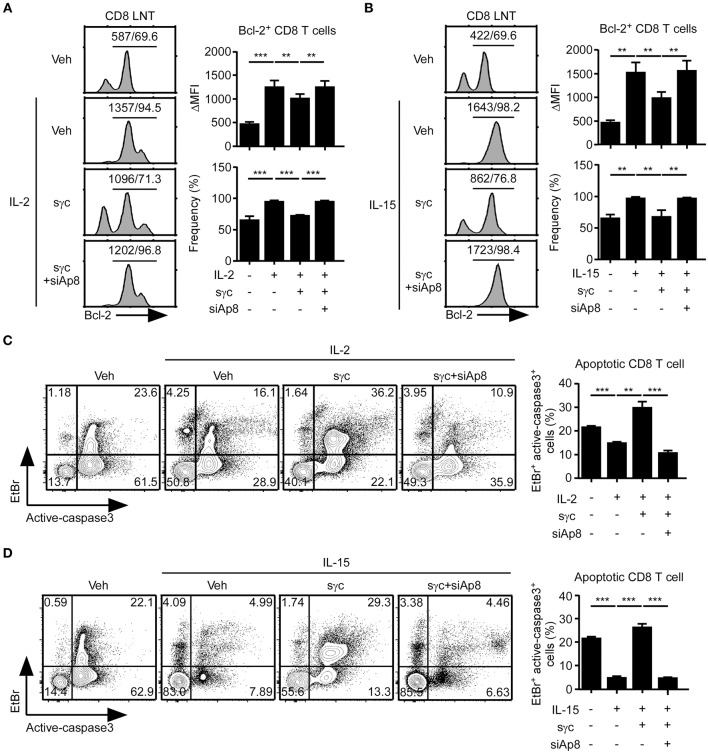
Effect of siAp8 in the IL-2 or IL-15 signaling. **(A,B)** Intracellular Bcl-2 expression in CD8^+^ LNT cells stimulated for 3 days with IL-2 **(A)** or IL-15 **(B)** in the presence of recombinant sγc, plus siAp8. Histograms show representative results from three independent experiments (left). Numbers on the solid line in the histograms indicates MFI and percentage gated on. Bar graphs show a summary of three independent experiments (Right, mean and SEM). **(C,D)** CD8^+^ LNT cell survival upon *in vitro* IL-2 **(C)** or IL-15 **(D)** stimulation in the presence of recombinant sγc, plus siAp8. Cell viability was determined by EtBr and active-caspase3 staining. Contour plots are representative of three independent experiments (left). Bar graphs show a summary of three independent experiments (right, mean and SEM). ^**^P < 0.01; ^***^P < 0.001. Veh, vehicle.

sγc interferes with IL-2 and IL-15 signaling in CD8^+^ T cells (6, 35, 36). Thus, we assessed the effect of siAp8 on IL-2 or IL-15 signaling in CD8^+^ T cells with anti-apoptotic Bcl-2 expression (Figures 4A,B). Bcl-2 expression upregulated by IL-2 (Figure 4A) or IL-15 (Figure 4B) was decreased in the presence of sγc and was rescued by siAp8 treatment (Figures 4A,B). Since reduced Bcl-2 expression is related to enhanced susceptibility to apoptosis and promoted caspase3 activity was indicative of increased cell death (37, 38), we further confirmed the effect of siAp8 with EtBr and active-caspase3 assay. Indeed, exogenous sγc promoted cell death (the EtBr^+^active-caspase3^+^ cells) in the presence of IL-2 (Figure 4C) or IL-15 (Figure 4D). siAp8 treatment, however, impeded the effect of sγc as shown by a significantly decreased frequency of the EtBr^+^active-caspase3^+^ cells (Figures 4C,D). These results indicated that siAp8 effectively suppresses the inhibitory functions of sγc in IL-2 and IL-15 signaling.

### siAp8 Ameliorates Pathogenesis of CIA

**Figure 5:**
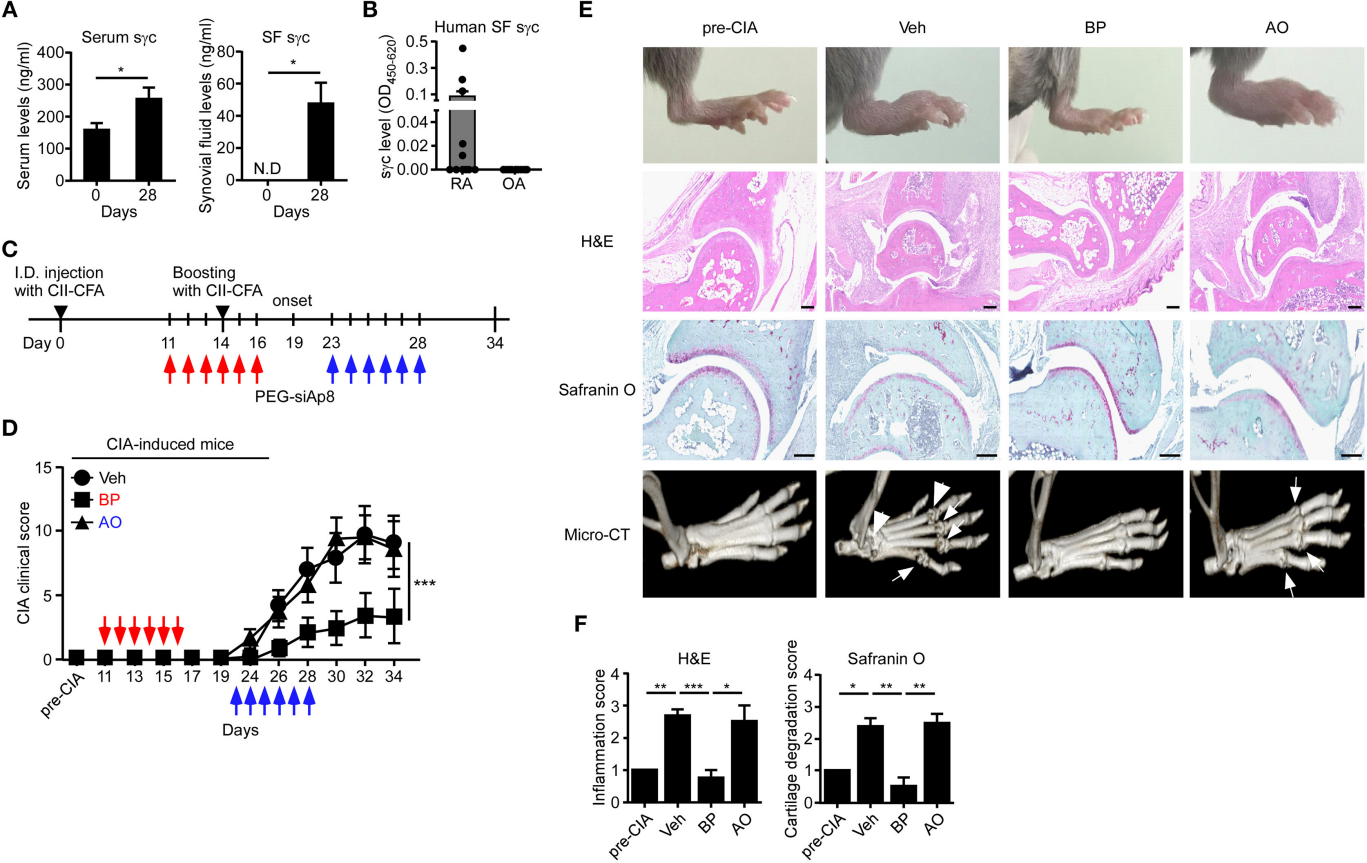
sγc expression in RA patients and CIA models, and effect of PEG-siAp8 in CIA progress. **(A)** Serums (left) or SF (right) were harvested at the indicated days and assessed for sγc by ELISA. **(B)** SF from patients of RA (*n* = 10) or OA (*n* = 9) were harvested and assessed for sγc by ELISA. **(C)** Injection of PEG-siAp8 to CIA mice were performed as shown. Red arrows indicate administration of PEG-siAp8 at BP, and blue arrows indicate administration of PEG-siAp8 at AO. **(D)** CIA was induced in DBA/1 WT mice as depicted in **(C)**. Administration points of PEG-siAp8 at BP or AO was indicated as red or blue arrows, respectively. CIA clinical scores are representative of two independent CIA experiments, each with five mice per group. **(E)** Representative photographs of the hind paws (Top). Representative joint tissue from the hind paws stained with haematoxylin and eosin (H&E) and with safranin O (Middle). Representative three-dimensional reconstruction of the hind paw assessed by micro-CT (Bottom). Arrows indicate destructed bones. **(F)** Bar graphs show a summary of two independent experiments of **(E)**, each with three mice per group (mean and SEM). ^*^P < 0.05; ^**^P < 0.01; ^***^P < 0.001. Veh, vehicle. Pre-CIA, Naïve DBA mice.

**Figure 6:**
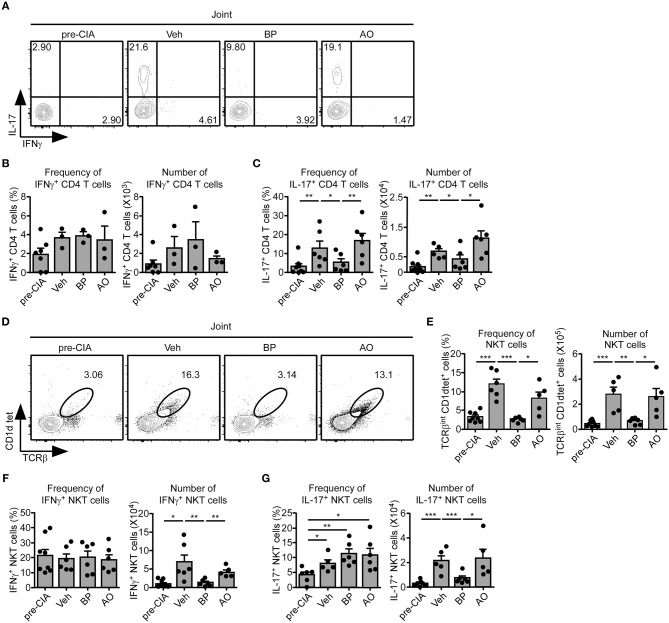
Effect of PEG-siAp8 in Th17 cells and NKT cells. The infiltrating cells were isolated from inflamed joints after 34 days of CIA induction. **(A–C)** IFNγ and IL-17 expressions of joint were assessed by intracellular staining. **(A)** IFNγ and IL-17 profiles are representative of two independent experiments. Bar graphs show a summary of the frequency and number of IFNγ^+^ **(B)** or IL-17^+^ **(C)** CD4^+^ T cells of two independent experiments, each with three mice per group (mean and SEM). **(D)** Contour plots of NKT cells in the inflamed joint are representative of two independent experiments. **(E)** Frequency and numbers of NKT cells are summarized. Bar graphs show a summary of the frequency (left) and number (right) of NKT cells of two independent experiments, each with three mice per group (mean and SEM). **(F,G)** IFNγ and IL-17 expressions of NKT cells were assessed. Bar graphs show a summary of the frequency (left) and number (right) of IFNγ^+^ **(F)** or IL-17^+^ **(G)** NKT cells of two independent experiments, each with three mice per group. ^*^P < 0.05; ^**^P < 0.01; ^***^P < 0.001. Veh, vehicle. Pre-CIA, Naïve DBA mice.

sγc is abundant in the serum and synovium of RA patients (23, 24). We confirmed that the sγc levels in CIA mice and RA patients are increased in the serum and SF (Figure 5A). sγc is expressed in SF of 50% of RA patients compared with that of OA patients. Among RA patients, three patients have high concentration of sγc and two patients have low concentration of sγc (Figure 5B and Table S2). Next, we administrated PEG-siAp8 to CIA mice at BP or AO, as depicted in Figure 5C. The injection of PEG-siAp8 at BP significantly ameliorated CIA progression with reduced joint swelling; however, similar CIA progress in mice treated with PEG-siAp8 at AO was observed as Veh control mice (Figures 5D,E top). According to the histological analysis, treatment of PEG-siAp8 at BP reduced infiltration of cells, cartilage damage, and bone erosion, whereas PEG-siAp8 at AO failed to prevent severe inflammation and cartilage damage (Figures 5E,F). In draining LN (dLN), there were no significant differences in frequencies of IFNγ^+^ or IL-4^+^ T cells, however, the frequency of IL-17^+^ T cells were notably increased (Figures S1A,B). Moreover, the frequencies of IFNγ^+^ or IL-17^+^ T cells in spleen (SP) were similar among all groups (Figures S1C,D). While frequency and number of IL-17^+^ T cells were increased in the inflamed joint of CIA mice with Veh or PEG-siAp8 at AO, the frequency and number were dramatically decreased in PEG-siAp8 at BP group (Figures 6A,C). PEG-siAp8 did not influence the frequency and number of IFNγ^+^ T cells (Figures 6A,B). To evaluate the involvement of NKT cells during CIA development, we checked NKT cells in SP and found that they were not different between naïve and CIA mice (Figures S2A,B). We profiled the subsets of NKT cells with T-bet vs. PLZF and found that PLZF^−^T-bet^+^ NKT cells were reduced after CIA induction and PLZF^−^T-bet^−^ NKT cells were increased after CIA induction (Figures S2C,D). In the inflamed joint, frequency and number of NKT cells were significantly increased in CIA mice with Veh or PEG-siAp8 at AO; however, the administration of PEG-siAp8 at BP efficiently decreased the frequency and number of NKT cells (Figures 6D,E). The frequency of IFNγ^+^ NKT cells was not different among the joint of all groups but the frequency of IL-17^+^ NKT cells was increased in all CIA mice compared with naïve mice (Figures 6F,G). Moreover, the numbers of IFNγ^+^ and IL-17^+^ NKT cells were enhanced in the inflamed joints of CIA mice with Veh or PEG-siAp8 at AO, but they were reduced by PEG-siAp8 at BP (Figures 6F,G). These results indicate that sγc has heterogeneous effects on CIA progress and the blocking point of IL-2 signaling is important in reducing the pathogenesis of CIA.

## Discussion

We investigated the mechanism of generating homodimeric sγc and the physiological roles of sγc in autoimmune disease, especially in RA. Unlike OA synovial tissues, the RA tissues contain high concentration of sγc (23). We demonstrated that sγc deficiency ameliorates clinical symptoms of EAE, Th17 cell-mediated autoimmune diseases (6). Since IL-17 is involved in the pathogenesis of RA (39), we expected that sγc would certainly play a role in autoimmune arthritis. We first identified a mechanism whereby the homodimeric sγc is formed by disulfide bonds between the Cys residues. Thus, we targeted the 9 a.a. to block the formation of homodimeric sγc with aptamers. We developed the most effective aptamer, siAp8, based on interrupting activity of dimeric formation and Th17 differentiation. Effect of the aptamer was confirmed in IL-2 and IL-15 signaling, as shown that their signals dampened by sγc are significantly restored by siAp8. To apply siAp8 to *in vivo* model, siAp8 was modified with 5'-PEG to maintain their circulation in the blood and to slow renal clearance (40), together with idT-3' to improve nuclease resistance (41). PEG-siAp8 treated at BP effectively improved clinical severity and pathology with decreased local Th17 cells and NKT cells in the joint. On the other hands, PEG-siAp8 applied at AO had no any beneficial effects on the pathogenesis of CIA. These different results based on time points of PEG-siAp8 administration implied that there may be a critical time point when sγc involves in the CIA pathogenesis. The podoplanin^+^ Th17 cells induce the formation of ectopic lymphoid-like structures (ELS) at inflamed sites of autoimmune diseases, such as RA (42), multiple sclerosis (43), systemic lupus erythematosus (44), and myasthenia gravis (45). ELS features the aggregates of T and B cells, formation of follicular DC networks with maintained or exacerbated disease severity (46). When disease is boosted in CIA model, autoreactive T cells are activated and then sγc levels increase in the inflamed joints. These environments at BP induce enhancement of Th17 differentiation, resulting in promotion of ELS formation and CIA progress. Thus, it was thought that CIA progress was dramatically prevented by blocking of sγc at BP, not at AO, since the function of sγc is blocked before Th17 differentiation and ELS formation. However, when ELS is already orchestrated in the inflammatory sites, γc cytokines are required for the growth of lymphocytes. Thus, we thought that blockade of sγc functions at AO would not affect ELS formation, resulting in induction of CIA progress. Further studies are required to confirm whether the formation of ELS is affected by sγc.

Role of NKT cells in RA is controversial. Although many studies indicated that NKT cells have beneficial effects in alleviating RA development (47–49), several studies reported that NKT cells have pathogenic roles in inducing severe symptoms of RA (19–21). In our results, disturbed sγc functions resulted in reduced frequency and number of NKT cells, especially IL-17^+^ NKT cells in the CIA joints. However, NKT cells in the spleen were not different between all CIA groups, implying that NKT cells are locally affected by sγc. One possible situation is that reduction of Th17 cells by impaired sγc would form suboptimal ELS, resulting in induction of suboptimal interaction between immune cells, including NKT cells and antigen presenting cells (APCs). Since interaction with APCs is important in activation of NKT cells (50), the suboptimal environment insufficiently activate NKT cells, resulting in ameliorating CIA development. Once ELS formation is established, the regulation of sγc function might be beyond its ability to manage CIA progress. Thus, suboptimal differentiation of Th17 cells in CIA mice with PEG-siAp8 treatment at BP would negatively affect activation of NKT cells through the impaired formation of ELS, indicating that sγc would be indirectly involved in differentiation of NKT cells. Since IL-2 promotes cytokine productions of NKT cell; however, IFNγ production is reduced in long-term exposure of IL-2, unlike IL-4, which is steadily enhanced (51), the direct involvement of sγc in activation of NKT cells could not be excluded. Moreover, NKT17 cells, not NKT1, developed properly without IL-15 (52, 53). Further study evaluating the detail mechanisms is required.

Based on the detection of both monomeric and dimeric sγc in the medium, the *in vitro* inhibitory effect of aptamer in dimeric formation of sγc and low capacity of aptamer in intracellular penetration (25), our results and previous report suggest that sγc is dimerized by disulfide bond presumably in the extracellular space or matrix. Although disulfide bond is catalyzed by protein disulfide isomerase (PDI) mainly in the endoplasmic reticulum (ER), previous studies showed that PDI is also secreted (54) and regulates disulfide bond modification of proteins, integrin (55, 56) and thrombin complexes (57, 58) in extracellular milieu. These reports reinforce our hypothesis about dimeric formation of sγc in extracellular environment. We do not exclude other mechanisms, but based on our findings and recent studies (55), we propose that dimeric sγc is formed by disulfide bond via catalytic activity of secreted PDI and the dimerization is inhibited by aptamer in extracellular space. Future studies on post-translational mechanisms of sγc are definitely required, because these results will provide us crucial information on the development of more effective regulators of sγc function.

In summary, our results demonstrated that homodimeric sγc is formed by Cys residues of the new 9 a.a. epitopes. We introduce a sγc-specific aptamer which blocked the formation of homodimeric sγc and successfully impaired its functions. Impaired sγc by siAp8 mitigated the progression of CIA with low differentiation of local Th17, NKT1, and NKT17 cells. Taken together, our results suggest that homodimeric sγc would be a critical pathogenic molecule in the progression of RA. Interestingly, we also identified such alternatively spliced sγc transcripts in human T cells (6), indicating an evolutionarily conserved mechanism of sγc expression and regulation. In fact, alternative splicing of γc in both humans and mice creates a C-terminal Cys residue that promotes the dimerization of sγc. Therefore, a blockade of homodimeric sγc formation and its function might be a novel clinical therapeutic approach to treat RA and other inflammatory autoimmune diseases.

## Author Contributions

CH designed research, analyzed data, and wrote the paper. BL performed experiments, analyzed data, and wrote the paper. YJ, GK, and LAA performed data acquisition and analysis. S-GL provided samples of RA patients and interpreted data. KK, ES, and SHR performed generation of aptamer and analyzed data. DHS provided critical review and interpretation of data. All authors have read and approved the final manuscript.

### Conflict of Interest Statement

The authors declare that the research was conducted in the absence of any commercial or financial relationships that could be construed as a potential conflict of interest.
