# Supplementary figures and images for: Specific Inhibition of Soluble γc Receptor Attenuates Collagen-Induced Arthritis by Modulating the Inflammatory T Cell Responses
Source: Front Immunol. 2019 Feb 8;10:209. doi: 10.3389/fimmu.2019.00209 (PMC6375885; doi:10.3389/fimmu.2019.00209)

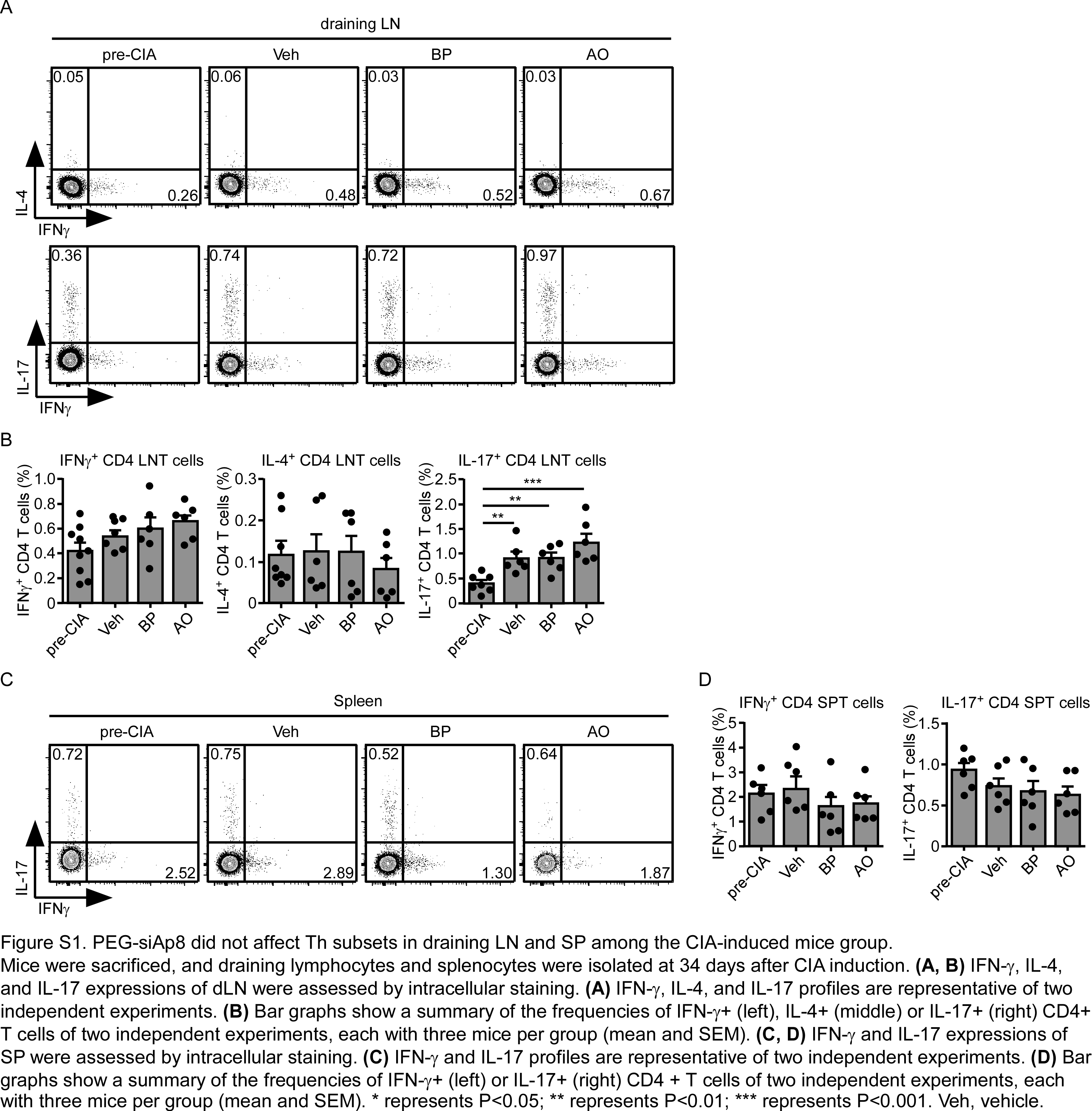

Supplement: Supplementary file 1 [file Image_1.TIF]

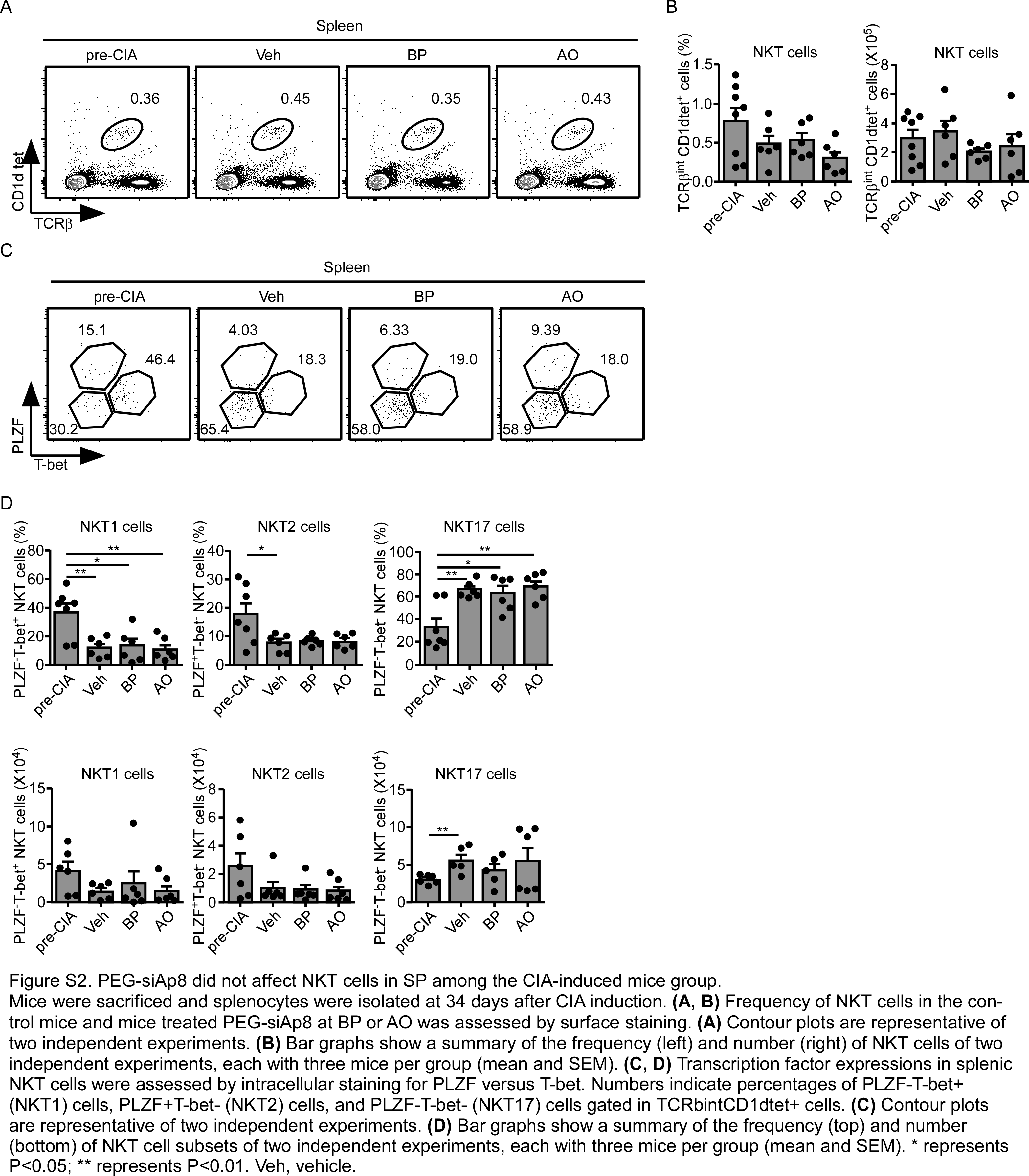

Supplement: Supplementary file 2 [file Image_2.TIF]

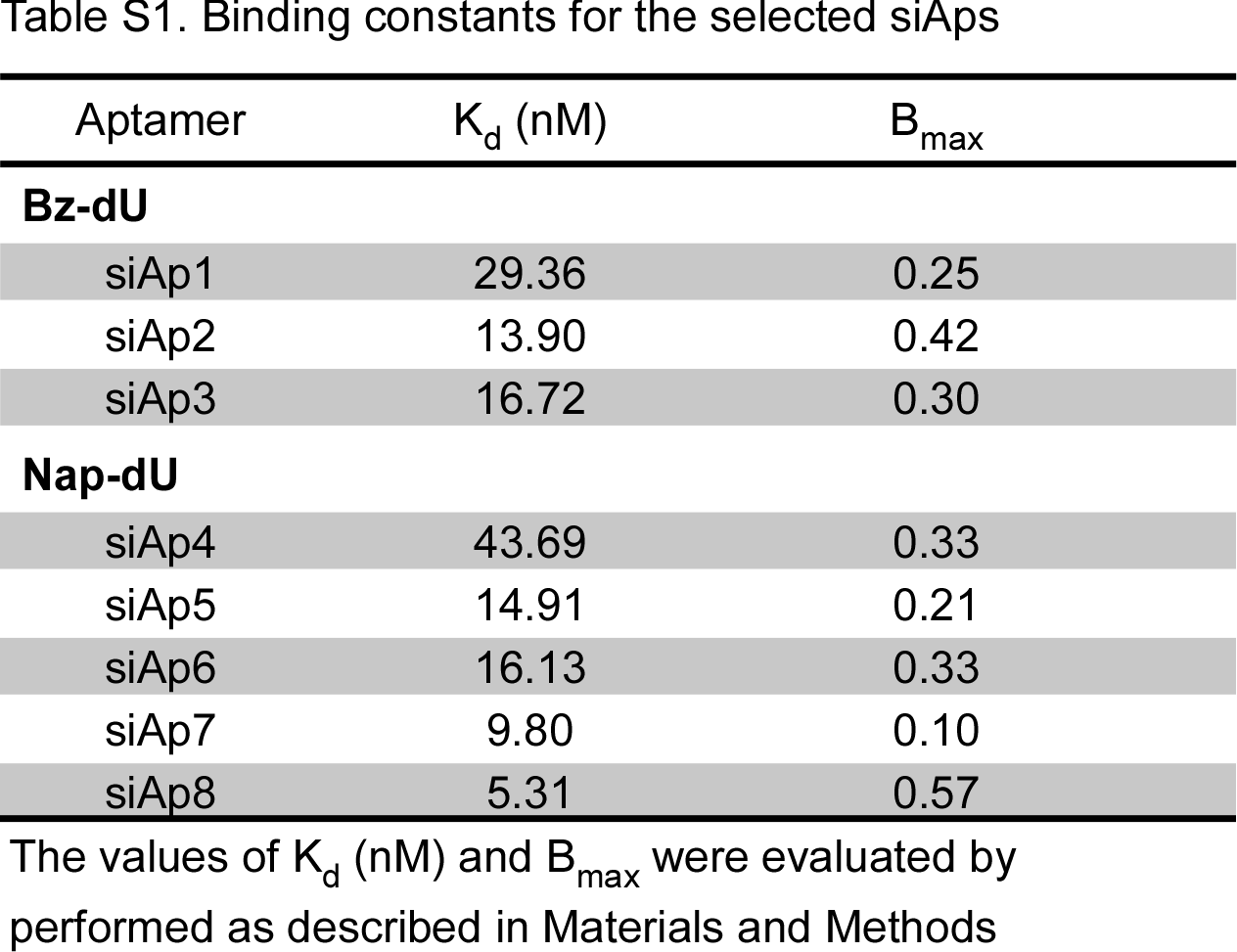

Supplement: Supplementary file 3 [file Image_3.TIF]

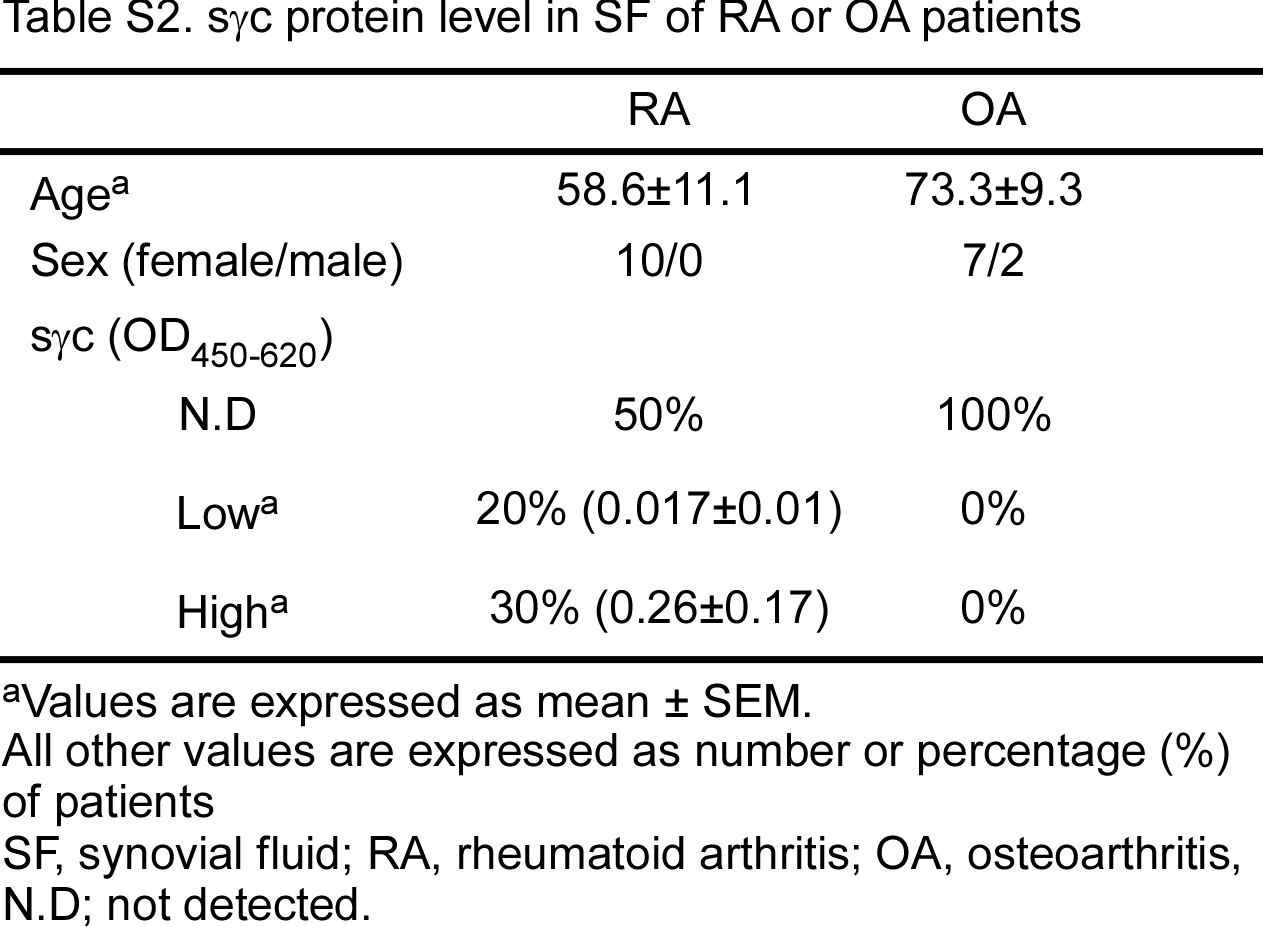

Supplement: Supplementary file 4 [file Image_4.TIF]
